# A Short Period of Darkness after Mixing of Growing Pigs Intended for PDO Hams Production Reduces Skin Lesions

**DOI:** 10.3390/ani10101729

**Published:** 2020-09-23

**Authors:** Lieta Marinelli, Paolo Mongillo, Paolo Carnier, Stefano Schiavon, Luigi Gallo

**Affiliations:** 1 Department of Comparative Biomedicine and Food Science, Università degli Studi di Padova, Viale dell’Università 16, 35020 Legnaro, Padua, Italy; lieta.marinelli@unipd.it (L.M.); paolo.carnier@unipd.it (P.C.); 2 Department of Agronomy, Food, Natural Resources, Animals and Environment, Università degli Studi di Padova, Viale dell’Università 16, 35020 Legnaro, Padua, Italy; stefano.schiavon@unipd.it (S.S.); luigi.gallo@unipd.it (L.G.)

**Keywords:** aggression, agonistic behavior, darkness, light, mixing, photoperiod, pig, regrouping, skin lesions, *Sus scrofa*

## Abstract

**Simple Summary:**

Mixing unacquainted growing pigs is a common practice in commercial herds to adjust the group size to the pen dimensions and to balance the body weights of pigs within pens. Aggressive behavior following regrouping may include fights that can result in skin lesions and detrimental economic effects. Strategies aimed at limiting such issues can therefore improve animal welfare in practice. In the present study, we investigated the effects of darkness, maintained for 48 h after the formation of new social groups, on the expression of agonistic behavior and on the accumulation of skin lesions of growing pigs. The provision of 48 h of darkness significantly reduced the number of skin lesions on the mid- and rear thirds of pigs’ body. However, no corresponding reduction was observed in agonistic behavior, suggesting that darkness decreases the efficacy of aggressions, rather than how often or for how long they are expressed. Furthermore, an analysis of the location of lesions indicates that aggressions towards a fleeing companion, rather than reciprocal ones, were those mostly affected by darkness. The present results identify in the provision of darkness an easily applicable and relatively inexpensive intervention, that leads to the reduction of one of the most problematic consequences of agonistic interactions, i.e., skin lesions.

**Abstract:**

Agonistic behavior after the regrouping of unfamiliar pigs has been recognized as one of the major welfare issues for pig husbandry, as it may result in lesions, lameness, and health problems. One scarcely investigated strategy to curb agonistic behavior is reducing the availability of visual stimuli potentially eliciting aggressions. In this study, we investigated the expression of agonistic behavior by growing pigs and the resulting accumulation of skin lesions over a period of 14 days following the formation of new social groups, which occurred in a condition of darkness maintained for 48 h. Compared to a simulated natural photoperiod (12 h light/day), darkness significantly reduced the number of skin lesions on the mid- and rear thirds of pigs’ body (*p* ≤ 0.01). A lack of corresponding decrease in frequency and duration of agonistic interactions suggests that darkness acts by decreasing the efficacy, not the expression, of aggressions. Furthermore, the location of lesions mostly affected by darkness indicates that the latter mostly acted by reducing the possibility of pigs to convey damage to a fleeing conspecific, rather than to one involved in a reciprocal fighting. The lighting regime provided did not affect growth performance traits of a 17-weeks feeding trial. The present results identify in the provision of darkness an easily applicable, and relatively inexpensive intervention, that leads to the reduction of skin lesions.

## 1. Introduction

Growing pigs in commercial herds are usually subjected to mixing events with unrelated and unacquainted animals [1]. This management procedure may occur several times from birth to slaughter [2] and generally aims at balancing the body weight of pigs within pens, in order to increase uniformity and to control stocking density by adjusting the group size to the pen dimensions [3]. Following regrouping, agonistic behavior may be intense, primarily serving to establish a new relative social ranking, thereby reducing future needs of disputes among animals [4]. Agonistic behavior can lead to fights, which result in skin lesions (SL), frequently used as a practical indicator of the extent and the severity of post-mixing aggressive behavior [5], as well as increased risk of lameness and of infections due to immunosuppressive effects [6]. Apart from the negative effects on animal welfare, the regrouping of unfamiliar pigs may also cause detrimental economic effects, negatively affecting growth rate and meat quality [3].

Agonistic behavior aimed at establishing relative social ranking in wild boars and feral pigs rarely ends with severe aggressions (i.e., bites), thanks to greater freedom of movement due to space allowance and fighting tactics based on complex and gradual behaviors [1,7]. Starting with reciprocal visual and olfactory inspection and gentle nudging, the interactions may escalate up to full-blown fights depending on signals indicating opponents’ intention to submit [8,9,10,11]. When the overt fight is started, the behavioral patterns mainly aim at minimizing the risk of being bitten and increasing the likeliness of biting. Accordingly, strategies at regrouping should reduce the number of interactions that escalate to aggressions or reduce the efficacy of aggressions in terms of reciprocal injuries. In this respect, several studies have investigated the efficacy of manipulating housing system, pen dimension, stocking density, and group size as methods to reduce aggressive consequences [1,3]. The introduction of environmental enrichment, which may increase the opportunity to carry out exploratory behavior and diverting pigs’ attention from conspecifics, has been explored [12], though with controversial results [1]. Other studies focused on the possibility to manipulate access to olfactory stimuli involved in the escalation of interactions by exposing animals to a diverse class of chemicals capable of altering olfactory information [13,14]. Much less attention has been paid to the possibility of manipulating visual signals and lighting regime; that has proven to affect growth rate and meat and ham quality of heavy pigs [15] and may be implicated in the escalation of inter-pig interactions.

To our knowledge, the manipulation of visual signals was only implemented by grouping animals after sunset [16,17], taking advantage of the lower phase of the circadian rhythm in a pig’s activity. In these conditions, the number of aggressions was reduced by about 50% in the 90 min following mixing, but this effect disappeared the following morning, resulting in a comparable number of SL after three days from regrouping. Although these results suggest that darkness only delays aggressions, the beneficial effects of reducing visual information at mixing might have been biased due to the duration of the treatment used. Indeed, the higher amount of fighting between pigs occurs in the first 24–48 h after regrouping [6], which suggests a possible advantage in applying darkness for longer than one night. Moreover, if darkness affects aggression by reducing availability of opponents’ visual information, extending its period of application could reduce outcome of aggressions (i.e., reciprocal injuries), regardless of their occurrence.

The aim of the present study was to deepen our knowledge on the effects of darkness at regrouping on aggressive behavior and skin lesions of growing pigs in a commercial farming situation.

## 2. Materials and Methods

### 2.1. Animals, Housing, and Experimental Design

The pigs involved in the present study are part of a larger feeding trial carried out at the research farm of the DAFNAE Department of the University of Padova, Italy [18,19,20]. Pigs were reared in accordance with EU Directive (2008/120/EC), and experimental procedures were reviewed and approved by the Ethical Committee for the Care and Use of Experimental Animals of the University of Padova, in accordance with the Italian legislation (D.Lgs. 26/2014, transposition of the EU 2010/63/UE directive).

Data were collected on 100 pigs (skin lesion counts) and 48 pigs (behavior observations)—specifically, barrows and gilts belonging to four different genetic types used in Italy for the production of heavy pigs to provide dry-cured hams. The piglets, who were born within the same week, arrived at the research farm around 80 d old and with a body weight (BW) close to 38 kg. At arrival, and until the beginning of the study, pigs were kept in four pens, homogeneous for genetic type (12 or 13 pigs/pen, two pens for each genetic type) and fed the same commercial diets according to the same feeding regime until the twenty-second week of age (86.5 ± 6.5 kg BW). Pens were located in two rooms (four pens/room), managed with the same ambient conditions and with an identical arrangement of pens within each room. Each pen was 5.8 × 3.8 m, had a slatted floor, and was equipped with a single-space electronic feeder (Compident Pig–MLP; Schauer Agrotronic, Prambachkirchen, Austria). Readers are referred to Schiavon et al. [18] for further details regarding the characteristics of pigs, their diets, growth performance, and carcass traits.

At the beginning of the study, the pigs were mixed, with the formation of new social groups, housed in the eight pens described above. Each of the eight groups was composed of 12 or 13 individuals (stocking density ≥ 1.6 m^2^/pig). Groups were balanced for BW, genetic types (e.g., each pen containing three or four pigs per genetic type), and sex (six or seven gilts and barrows per pen, and one or two of each sex, for each genetic type).

**Figure 1:**
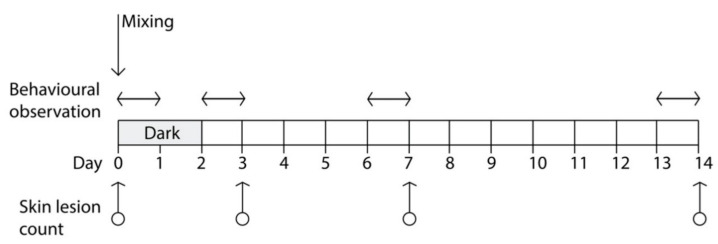
Schedule of skin lesion counting (on eight pens, *n* = 100 pigs) and behavior observations (on four pens, *n* = 48 pigs), relative to the day of mixing (Day 0); darkness was applied to half of the pens for the first 48 h after mixing (grey area).

During mixing procedures (Day 0) and for the subsequent 48 h, a lighting regimen of darkness was kept in one of the two rooms (Figure 1), thus affecting half of the pigs (*n* = 50) in the study; darkness was obtained by covering the windows, the only source of ambient light in the room, with a fourfold black plastic polypropylene sheets. Simulated natural photoperiod (12 h light/day) was reintroduced in this room at h 10:00 of Day 2 and maintained for the rest of the experiment; that ended after two weeks at Day 14. In the other room, a lighting regimen of 12 h light/day was kept for the entire duration of the experiment (windows were covered in this room as well to obtain precise control on the photoperiod). Apart from the lighting regimen in the first 48 h following mixing procedures, pigs of the two rooms were managed and fed in the same way.

### 2.2. Skin Lesion Counts

The total number of SL was recorded for each pig immediately before mixing (Day 0), after 72 h post-mixing (Day 3), 24 h after the natural photoperiod was reintroduced in the darkened room, and at Day 7 and Day 14 following regrouping (Figure 1). Lesions were recorded by direct observation of each pig, and it was performed independently by two trained observers who counted the total number of fresh lesions. A lesion was defined as a single and continuous scratch, regardless of severity, whereas freshness was judged on the basis of lesion color, usually red-pink, and development of scabbing [21,22]. Lesions were recorded independently on three different locations of the body: front (head, neck, shoulders, and front legs), middle (flanks and back), and the rear part of the animal (rump, hind legs, and tail), because the accumulation of skin lesions in different locations has been associated with different types of agonistic behavior [5,22].

### 2.3. Behavioral Observations

Infrared cameras, capable of recording in the dark, were installed overhead (Bullet IP cam, Y-cam Solutions Ltd., London, UK). Each camera framed the area occupied by one pen. A total of four cameras were installed, two for each room. Therefore, behavioral data were collected from a subsample of 48 animals, equally balanced between those subjected to darkness, and those kept in a 12 h photoperiod. Unfortunately, it was not possible to identify pigs of different genetic type or gender on videos, so these effects were not considered in the statistical models in which behavioral observations were analyzed.

To avoid interference due to the presence of operators performing the SL count, behavioral data were collected during the 24 h preceding each SL count. The only exception was represented by the behavioral data collected in the first 24 h following the regrouping of pigs, which occurred the day after the first (baseline) SL count. Collection of behavioral data was performed with the Observer software (ver. 12, Noldus Inc., Groeningen, The Netherdlands) on videos recorded on the following days of the trial (Figure 1):

- First session: from 10:00 a.m. (immediately after the end of mixing procedures) to 9:59 a.m., of Day 1;
- Second session: from 10:00 a.m. of Day 2 (immediately after reintroduction of the normal photoperiod in the pens that received the dark lighting regimen) to 9:59 a.m. of Day 3;
- Third session: from 10:00 a.m. of Day 6 to 9:59 a.m. of Day 7;
- Fourth session: from 10:00 a.m. of Day 13 to 9:59 a.m. of Day 14 (end of the trial).

Observations were performed in one interval of 10 min for each of the 24 h of observation days, resulting in a total of 24 intervals per day of observation and 96 intervals per pen for the whole study. During each observation interval, the expression of agonistic behavior by individual pigs was recorded with a focal animal, continuous sampling technique [23]. Agonistic behavior was defined as any interaction involving bites, attempts to bite, physical contact aimed at displacing, or putting off balance another pig (e.g., by pushing one’s body against the other, or by putting one’s muzzle under the body of the other pig). When such agonistic interaction lasted less than 1 s (i.e., one bite or one attempt to bite not followed by any other agonistic behavior), they were recorded as point events (AGO < 1 s). When the interactions lasted more than 1 s (AGO > 1 s), their duration was recorded, resulting in both number and duration of these interactions being used in statistical analysis.

Since the recording of agonistic behavior was performed for each pig in the pen, an interaction involving only two pigs would result in two recorded episodes of agonistic behavior of identical duration. For interactions involving more than two animals, as many episodes were recorded, as was the number of animals involved; the duration of such episodes, however, could be different since subjects could be involved for a different time in the same interaction (e.g., pig A starts an agonistic interaction with pig B, then pig C is involved along with A and B, then B stops interacting, leaving A and C to continue the interaction; in this case, three episodes of different duration would be recorded).

In addition to agonistic behavior, the number of animals in quadrupedal stance at the beginning of the 10 min observation interval was recorded as a measure of overall activity in the pen within the observation interval (STAND).

### 2.4. Performance Traits

In order to evaluate possible long-term effects of the darkness condition provided in the first 48 h following mixing, the main performance traits recorded for pigs in the feeding trial were taken into account. To this purpose, individual BW was collected at the start and at the end of the feeding trial for all the animals (100 pigs and eight pens), whereas individual feed intake (ADFI) was measured daily through the electronic feeder [18]. Data collected were used to compute individual average daily gain during the feeding trial (ADG), average individual daily feed intake, and individual feed efficiency, expressed as gain to feed ratio (G:F).

### 2.5. Editing and Statistical Analysis

Total SL count was computed for each pig by summing the SL recorded on the three different locations of the body. Behavioral observations were partitioned in two classes according to the daytime hours in which they occurred (DH: 08:00 to 19:59 h and 20:00 to 07:59 h), which also corresponded to the time at which lights were turned on/off, respectively, with the exception of the first 48 h in the pigs subjected to the dark lighting regimen. After a preliminary analysis aimed at examining their approximation to the normal distribution, data about the SL and the number of agonistic interactions were log transformed prior to statistical analysis.

Log of SL counts were analyzed according to a linear mixed model (SAS 9.4, SAS Institute, Cary, NC, USA) that included the fixed effects of sex (two levels), genetic types (four levels), lighting regime in the first 48 h following mixing (LR, two levels), days of the trial (DAY, four levels), operator (two levels), the DAY × LR interaction, and the random effects of pen within LR and of animal within genetic type and sex. Polynomial contrasts were estimated between the least square means of DAY to examine the response curve of each trait (linear, quadratic, and cubic components) with the advancing of time after mixing. Contrasts between least squares means of LR were estimated separately within each DAY for traits where the LR × DM interaction was significant (*p* < 0.05).

Data concerning the logarithm of the number of AGO < 1 s and AGO > 1 s, the total and mean duration of AGO > 1 s, and the incidence of STAND were analyzed according to a linear mixed model (SAS 9.4, SAS Institute, Cary, NC, USA) that included the fixed effects of LR, days from mixing (DM, four levels), daytime hours (DH, two levels) and the LR × DM and LR × DH interactions, and the random effects of hour of observation within DH and of pen within LR. Polynomial contrasts were estimated between the four least square means of DM to examine the response curve of each trait (linear, quadratic, and cubic components) with the advancing of time after mixing. Contrasts between least squares means of LR were estimated separately within each DH for traits where the LR × DH interaction was significant (*p* < 0.05).

Last, performance traits were analyzed according to a linear mixed model (SAS 9.4, SAS Institute, Cary, NC, USA) that included the fixed effects of sex (two levels), genetic types (four levels), diet (four levels), and LR, and the random effect of pen within LR and diet.

## 3. Results

### 3.1. General Statistics and Skin Lesions Count

Descriptive statistics of main performance traits recorded on pigs during the whole feeding trial, of SL counts, and of behavioral observation traits during the two weeks following pigs’ regrouping are given in Table 1.

At the beginning of the feeding trial, when the pigs were moved to new pens and regrouped, the average BW of pigs approached 87 kg. At the end of the feeding trial, after around 17 weeks on feed, the BW of pigs averaged around 165 kg, which is the target weight for heavy pigs aimed to dry-cured ham production. The ADG was close to 0.66 kg/d and, given an ADFI around 2500 g/d from the start to the end of the trial, the average F:G ratio approached 3.85. The coefficient of variability was below 10% for all performance traits considered except for growth rate, which showed a coefficient of variation close to 14%.

At the fourteenth day following the regrouping procedure, an average total number of 5.24 skin lesions was recorded, with 56% of them being located in the front body area, whereas the middle and rear body area showed a similar SL count distribution. Variation in SL count was high for all the locations in the body.

The average number of AGO > 1 s in the 10 min/h of observation interval approached 2.59, with an average mean duration close to 7 s, whereas on average, 17% of pigs in the pen were standing at the beginning of the 10 min of the observation interval. Additionally, for the behavioral observations, the variation observed was very large.

Average accumulation of SL in the two weeks following pigs regrouping was not influenced by the lighting regime adopted in the first 48 h from mixing (Table 2) independently from the body area. Conversely, the day of trial significantly influenced the distribution of SL irrespective of their position in the body (*p* < 0.0001). As a general trend, the change of log of SL with the advancing of days after regrouping followed a quadratic (SL evaluated in the flanks and back and total sum of SL counts) or cubic (SL evaluated in the front or in the rear part of the body) pattern. Namely, log of SL increased from the day of mixing (Day 0 was considered as a baseline reference for SL) until Day 3 (head, neck, shoulders, and front legs) or 7 from mixing (middle and rear part of the body and total sum of SL counts), whereas it decreased thereafter in all positions of the body.

**Figure 2:**
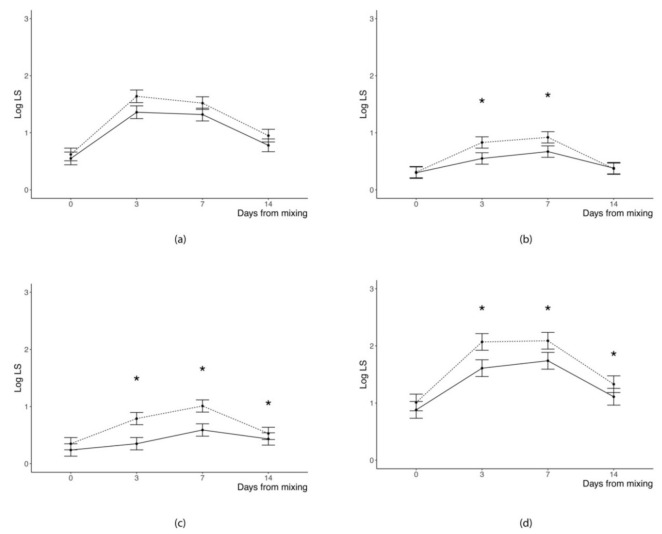
Least squares means of logarithm of skin lesions count assessed in: (**a**) front, (**b**) middle, and (**c**) rear position and of (**d**) logarithm of sum of counts at different days from mixing (Days Mix) for pigs kept in dark rooms (solid line) or with a 12 h photoperiod (dotted line) during the first 48 h after mixing (*p < 0.05 for difference between means of the two groups within any given day from mixing).

Moreover, we found a significant (*p* ≤ 0.01) LR × DAY interaction for all the SL counts except for those in the front body area; this suggests that the dynamic of SL accumulation was different according to the lighting regime provided to the pigs during the first 48 h since regrouping. Indeed, pigs kept in the dark room during the first 48 h since regrouping showed a similar pattern of variation since the day of mixing but lower SL accumulation 3 and 7 d following mixing, compared to those pigs provided with a 12 h photoperiod for the whole trial (Figure 2). Differences in SL accumulation between pigs of the two groups were no longer evident 14 d after mixing, with the only exception of skin lesions in the rear part of the body. 

### 3.2. Behavioral Observations

Table 3 reports the least squares means of the agonistic behavior variables and of the overall activity, as a function of lighting regimen, days of trial, and daytime hours, as well as the LR × DM and LR × DH interactions on the same variables. No effect was found for the LR or its interaction with DAY on any of the agonistic behavioral variables or the overall activity.

There were more AGO < 1 during daytime than at night (*p* = 0.001); no change was observed in the number of such interactions as a function of DAY.

There were more agonistic interactions longer than 1 s during daytime than at night, and they were also longer, in terms of average as well as total duration. The mean and total duration of such interactions decreased with increasing days from mixing, and fewer of such interactions occurred as the days from mixing increased.

The incidence of standing pigs on total number of pigs in the pen was affected in a similar manner, with a higher overall activity during daytime than at night, and a decrease of overall activity as a function of days from mixing.

### 3.3. Performance Traits

**Figure 3:**
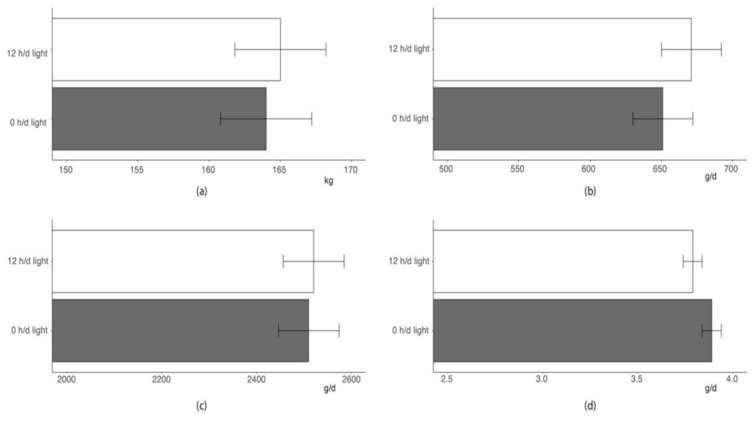
Least squares means of: (**a**) final BW, (**b**) overall average daily gain, (**c**) average daily feed intake, and (**d**) feed-to-gain ratio of pigs given 0 h (grey bar) or 12 h light daily (white bar) during the first 48 h after mixing (*p* value of the differences between means > 0.10).

As shown in Figure 3, the lighting regime during the first 48 h following regrouping did not affect growth performance traits of a 17-weeks feeding trial (*p* > 0.10). Pigs of two groups showed similar BW at the end of the feeding trial and comparable growth rate. As average feed intake was nearly identical in pigs provided with 48 h dark or normal photoperiod after mixing, the feed-to-gain ratios of the pigs of the two groups were also very similar.

## 4. Discussion

Agonistic behavior expressed when unfamiliar pigs are housed into newly formed social groups has been recognized as a one of the major welfare issues for pig husbandry [1]. Although aggression following mixing of growing or breeding pigs has been widely investigated in the last decades, mitigation strategies proposed seem scarcely implemented in commercial herds, so that the problem has not been reduced yet [3,4]. In the present study, we investigated if the provision of darkness in the 48 h after the formation of new social groups in growing pigs is effective in reducing the expression of agonistic behavior, and its outcome in terms of accumulated skin lesions.

Skin lesions are a useful and practical indicator of aggressive behavior and poor welfare, as their assessment requires little time, no specific equipment, and limited training [22]. Moreover, SL are a direct outcome of physical damages due to agonistic interactions, and their frequency and intensity can be related to the level of welfare impairment following regrouping.

In this study, a large variation in the total number of SL was found, similar to Turner et al. [5]. In agreement with the latter and other studies [5,9,24], lesions were mostly concentrated in frontal part of the body. Turner and collaborators [5] suggested that lesions in such region are the outcome of reciprocal active fighting, whereas those accumulated in the medium and rear part of the body are correlated to receiving aggression and retreating.

The accumulation of SL progressively increased over time, from the third to the seventh day of trial for front SL and middle, rear, and total ones, respectively. This pattern is generally consistent with data from the literature, although the schedule of SL evaluation after regrouping is variable. Turner and collaborators [5] observed an increase of SL 24 h post-mixing in the vast majority of pigs evaluated, with a greater increase for front SL compared to the rear body part. Similarly, Wurtz and collaborators [22] reported a steep increase of SL in all body locations 24 h following regrouping, whereas three weeks after regrouping SL decreased and accumulation was generally lower than that scored at regrouping. Stukenborg and collaborators [24] observed an increase in SL evaluated 48 h after regrouping on the front part of the body on the majority of pigs considered; conversely, no positive difference was found for over 60% and 70% of pigs in LS observed in the middle and rear regions, respectively, concluding that their contribution in the prediction of agonistic behavior seemed to be rather insignificant. Differences in the trend of variation of SL found in the present study could corroborate such conclusion, as front SL peaked 3 d after mixing, including the period when agonistic behavior is particularly intense, whereas SL judged at the medium and rear parts of the body peaked at later times.

Keeping pigs in the dark during the first 48 h since regrouping, compared to those provided with a 12 h photoperiod for the whole trial, did not affect the average accumulation of SL over the two weeks following regrouping or the pattern of variation of SL since the day of mixing; however, it significantly decreased the sum of SL and the accumulation of SL in the medium and rear part of the body in the first 7 d following regrouping, thus mitigating the negative effects of mixing unfamiliar pigs on skin integrity. Barnett and collaborators [16] observed a short-term decrease of aggression in pigs mixed 30 min after sunset compared to pigs mixed in the morning. However, the accumulation of SL three days post-mixing was similar between groups, suggesting that mixing after sunset simply delayed SL rather than really mitigating the effects of regrouping.

With regard to agonistic behavior, its expression was mostly affected by the time of day, with both duration and frequency of agonistic interactions being fourfold higher during daytime than nighttime. Our results are in agreement with previous studies reporting higher peaks in the expression of agonistic interactions in the middle of the day and lower peaks at night following a circadian rhythm [24]. In the present study, this pattern was clearly associated with the pigs’ overall activity level, which was also, predictably, lower at night than during daytime. It is worth noting, however, that no difference in the effect of daytime on agonistic behavior or activity levels was found between the two experimental groups. Therefore, the provision of 48 h of darkness did not alter pigs’ circadian rhythm, at least in terms of behavior. Although the timing by which photoperiod acts on agonistic behavior has not been investigated before, other physiological traits, such as melatonin concentrations, have been shown to closely follow abrupt changes in photoperiod; nonetheless, even melatonin secretion may take up to one week to fully entrain after a new photoperiod is instated [25]. Thus, our findings are well consistent with a lack of relevant effects in the provision of 48 h of darkness on circadian rhythm.

Agonistic behavior was also affected by the time elapsed from the day of mixing. The effect was clearly evident on the average and total duration of agonistic interaction, which decreased linearly (for average and total duration) or along a quadratic curve (average duration only), from Day 0 to the fourteenth day after mixing. Additionally, the number of agonistic interactions longer than 1 s decreased linearly with the advancing of days of trial, whereas no significant effect of day from mixing was observed on the number of short, instantaneous agonistic events. Therefore, although agonistic behaviors do not cease to occur, they appear to be solved significantly faster over time [26]. Therefore, our results confirm that aggressive behaviors aimed at re-establishing a social hierarchy in the new groups mostly occur during the first 48 h after mixing, as previously reported [6].

Previous studies suggested that impeding access to visual stimuli when groups of unacquainted pigs are newly formed may reduce the expression of agonistic behavior [16]. However, as the aggressions resume at the re-establishment of visibility (end of darkness period), this practice merely delayed the expression of this behavior [16]. In our experiment, the expression of agonistic behavior was not affected by the provision of 48 h of darkness, nor by its interaction with the time from mixing, indicating that agonistic interactions were concentrated during the first days after mixing, and progressively decreased over time, in both groups. This implies that there was no rebound in the frequency or duration of agonistic interactions in animals that had been kept in darkness once visibility was re-established. Our results are in contrast with those reported by Barnett et al. [16], who observed increased agonistic interactions between pigs being mixed before a 12 h (overnight) of darkness as the end of this period coincided with the peak of expression of agonistic behaviors within the group of pigs. On the contrary, in our study, this peak occurred while pigs were still kept in the dark, indicating that the provision of a 48-h period of darkness is sufficient to overcome such an acute agonistic phase; at the same time, the period is long enough to avoid relapses when visual stimuli are again made available. A further implication of the lack of differences between experimental groups in the expression of agonistic behavior over time is that the removal of visual stimuli of conspecifics does not seem to prevent pigs from engaging in agonistic interactions, nor to carry them on for as long as in plain visibility. Thus, at least in the conditions assessed by the present study, other sensory modalities seem sufficient to drive pigs’ agonistic behavior. Again, this is in line with previous literature, showing not only a significant role of olfactory [27,28,29,30] and pheromonal perception [14,31,32] on agonistic behavior, but also that removal of visual stimuli (e.g., by temporarily blinding the animals, or by covering their heads with hoods) brings no alteration in pigs ability to form and maintain a hierarchy [33,34].

At first glance, the significant effect of the provision of darkness in the first days after regrouping on the accumulation of SL stands at odds with a lack of corresponding effects on the frequency and duration of agonistic behavior. On a closer look, however, the two findings are quite informative of the mechanism by which darkness affected pigs’ agonistic behavior. While the lack of availability of visual stimuli did not seem to be a relevant factor in eliciting agonistic behavior, it did impact pigs’ abilities to accurately aim at the opponent and inflict damage. It should be further noted that the difference in SL between experimental groups was limited to the mid- and rear body regions, rather than the frontal ones. As discussed above, the differential location of SL is thought to reflect a different role of the animal in the agonistic interaction [5]. In this sense, the lack of access to visual stimuli, rather than impacting the efficacy of close-contact, reciprocated fights, seems to have reduced the possibility of pigs to convey damage to a fleeing companion.

Last, managing photoperiod in the first 48 h following regrouping did not affect long-term performance of pigs. Indeed, pigs of the two groups evidenced comparable growth rate and feed efficiency and ended the 17 weeks of finishing at similar BW.

## 5. Conclusions

In this study, we showed that provision of 48 h of darkness in newly formed groups of fattening pigs significantly reduces the accumulation of skin lesions, particularly on the mid- and rear thirds of pigs’ bodies. A lack of corresponding decrease in frequency and duration of agonistic interactions indicates that darkness acts by decreasing the efficacy of aggressions. Furthermore, the location of lesions mostly affected by darkness indicates that the effect was specific to aggressions towards a fleeing subject. The present findings have both theoretical and practical relevance. On the one hand, they shed light on the (limited) role of visual stimuli in eliciting agonistic behavior in pigs. They also provide suggestive evidence that an abrupt alteration of photoperiod does not bear a relevant impact on pig circadian rhythms, at least for what concerns overall activity levels and agonistic behavior. On the other hand, the results identify in the provision of darkness an easily applicable and relatively inexpensive intervention that leads to the reduction of one of the most problematic consequences of agonistic interactions, i.e., skin lesions. Certainly, some aspects will have to be addressed in further studies. For instance, it would be important to determine if the positive effects of darkness would also be observed in different categories of pigs than the ones assessed in this study (e.g., younger animals, intact males, pigs belonging to other genetic types). Moreover, it would be interesting to assess whether longer extension of the darkness period would exert more profound effects on the expression of agonistic interactions or their outcomes. However, considering the relatively quick effects that abrupt changes may have in the circadian rhythm in pigs, it is possible that longer exposures to darkness would have a larger effect, including possibly undesirable consequences on their welfare or growth.

## Figures and Tables

**Table 1:** Descriptive statistics of main performance traits of growing pigs and of skin lesions count, number of agonistic interactions shorter and longer than 1 s (AGO > 1 s), total and mean duration of AGO > 1 s, and incidence of standing pigs on total number of pigs in the pen (STAND).

Item	No	Mean	SD	Min	Max
Main performance traits of pigs:					
Initial body weight, kg	100	86.5	6.9	66.4	103.7
Final body weight, kg	96	164.4	13.4	131.0	196.7
Average daily gain, kg/d	96	660	94	428	844
Average daily feed intake, g/d	96	2515	236	1696	2903
Feed to gain ratio	96	3.85	0.35	3.29	4.77
Counts of skin lesions assessed on:					
Front, no	798	2.98	3.58	0	29
Middle, no	798	1.21	2.01	0	17
Rear, no	798	1.04	1.54	0	12
Sum of skin lesions count, no	798	5.24	5.84	0	40
Agonistic interactions shorter than 1 s, no	383	1.78	3.42	0	26
Agonistic interactions longer than 1 s, no	383	2.59	5.16	0	38
Total duration of AGO > 1 s, s	114	56	99	2	649
Mean duration of AGO > 1 s, s	114	6.9	6.4	1	38
STAND, %	383	16.7	20.1	0	100

**Table 2:** Least squares means of logarithm of skin lesions count (SL) for lighting regimen during the first 48 h after mixing and days of trial.

Item	Body Location			Log of Sum of SL
	Front	Middle	Rear	
Lighting regimen (LR)				
0 h/d light	1.00	0.47	0.36	1.33
12 h/d light	1.18	0.61	0.67	1.63
SEM	0.10	0.09	0.10	0.14
*p* value	>0.10	>0.10	0.07	>0.10
Days of trial (DAY)				
0	0.59	0.31	0.30	0.94
3	1.50	0.69	0.57	1.84
7	1.42	0.79	0.80	1.92
14	0.86	0.37	0.40	1.22
SEM	0.08	0.07	0.08	0.10
*p* value of effect	<0.0001	<0.0001	<0.0001	<0.0001
*p* value of contrasts:				
Linear	<0.0001	0.05	0.0002	<0.0001
Quadratic	<0.0001	<0.0001	<0.0001	<0.0001
Cubic	0.01	>0.10	<0.0001	>0.10
LR × DAY				
*p* value	>0.10	0.01	0.001	0.01

**Table 3:** Least squares means of the number of agonistic interactions shorter (AGO < 1 s) and longer than 1 s (AGO > 1 s), of the total and mean duration of AGO > 1 s, and of the incidence of standing pigs on total number of pigs in the pen (STAND) for lighting regimen during the first 48 h after mixing, session of observation, and daytime hours ^1^.

Item	AGO < 1 s, log	AGO > 1 s, log	Duration of AGO > 1 s, s		STAND, %
			Total	Mean	
Lighting regimen (LR)					
0 h/d light	0.64	0.73	39	6	17.3
12 h/d light	0.57	0.64	41	5	16.2
SEM	0.14	0.14	20	2	2.6
*p* value	>0.10	>0.10	>0.10	>0.10	>0.10
Session of observation (DAY)					
First (day of trial 0 to 1)	0.70	0.88	103	9	20.3
Second (day of trial 2 to 3)	0.53	0.66	39	5	13.8
Third (day of trial 6 to 7)	0.54	0.60	4	5	15.9
Fourth (day of trial 13 to 14)	0.65	0.58	13	5	16.7
SEM	0.13	0.13	19	1	2.7
*p* value of effect	>0.10	0.06	<0.0001	0.007	0.05
*p* value of contrasts:					
Linear		0.01	<0.0001	0.01	>0.10
Quadratic		>0.10	0.02	0.07	0.03
Cubic		>0.10	>0.10	>0.10	>0.10
Daytime hours (DH)					
08:00 to 19:59 h	0.86	1.02	65	8	24.1
20:00 to 07:59 h	0.35	0.34	15	3	9.3
SEM	0.13	0.14	17	1	8.0
*p* value	0.001	<0.001	0.02	<0.001	<0.001
LR × DAY					
*p* value	>0.10	>0.10	>0.10	>0.10	>0.10
LR × DH					
*p* value	>0.10	0.07	>0.10	>0.10	>0.10

^1^ Agonistic behavior and activity measured during the first 10 min of each hour.
